# Spike Firing and IPSPs in Layer V Pyramidal Neurons during Beta Oscillations in Rat Primary Motor Cortex (M1) *In Vitro*

**DOI:** 10.1371/journal.pone.0085109

**Published:** 2014-01-20

**Authors:** Michael G. Lacey, Gerard Gooding-Williams, Emma J. Prokic, Naoki Yamawaki, Stephen D. Hall, Ian M. Stanford, Gavin L. Woodhall

**Affiliations:** 1 School of Clinical and Experimental Medicine (Neuronal Networks Group), College of Medical and Dental Sciences, University of Birmingham, Birmingham, United Kingdom; 2 Aston Brain Centre, Aston University, School of Life and Health Sciences, Birmingham, United Kingdom; University of Alberta, Canada

## Abstract

Beta frequency oscillations (10–35 Hz) in motor regions of cerebral cortex play an important role in stabilising and suppressing unwanted movements, and become intensified during the pathological akinesia of Parkinson's Disease. We have used a cortical slice preparation of rat brain, combined with concurrent intracellular and field recordings from the primary motor cortex (M1), to explore the cellular basis of the persistent beta frequency (27–30 Hz) oscillations manifest in local field potentials (LFP) in layers II and V of M1 produced by continuous perfusion of kainic acid (100 nM) and carbachol (5 µM). Spontaneous depolarizing GABA-ergic IPSPs in layer V cells, intracellularly dialyzed with KCl and IEM1460 (to block glutamatergic EPSCs), were recorded at −80 mV. IPSPs showed a highly significant (P< 0.01) beta frequency component, which was highly significantly coherent with both the Layer II and V LFP oscillation (which were in antiphase to each other). Both IPSPs and the LFP beta oscillations were abolished by the GABA_A_ antagonist bicuculline. Layer V cells at rest fired spontaneous action potentials at sub-beta frequencies (mean of 7.1+1.2 Hz; n = 27) which were phase-locked to the layer V LFP beta oscillation, preceding the peak of the LFP beta oscillation by some 20 ms. We propose that M1 beta oscillations, in common with other oscillations in other brain regions, can arise from synchronous hyperpolarization of pyramidal cells driven by synaptic inputs from a GABA-ergic interneuronal network (or networks) entrained by recurrent excitation derived from pyramidal cells. This mechanism plays an important role in both the physiology and pathophysiology of control of voluntary movement generation.

## Introduction

Beta oscillations (15–35 Hz) are a characteristic feature of neuronal network activity in primary motor cortex (M1) and such activity has been suggested to reflect an idling state of cortex, which prevails in the absence of appropriate sensory input [1]. However, other studies [2] have indicated that motor cortical beta activity may reflect active inhibition of movement and therefore likely to be involved in maintaining postural tone [3]. This latter aspect has relevance for the dopamine-depleted state, as seen in Parkinson's disease (PD), where beta activity within cortical-subcortical motor loops is abnormally enhanced [4], [5], [6] and which coincides with the emergence of movement disorders [7] such as akinesia and bradykinesia. Administration of levodopa or deep brain stimulation of the subthalamic nucleus appears to reduce this coherent beta frequency activity, which is accompanied by motor improvement [8], [9]. Similar effects can be seen with antidromic stimulation of deep motor cortical pyramidal cells [10], suggesting that M1 itself is important in the pathogenesis and/or treatment of PD and recent advances using optogenetic approaches have shown that afferent axons projecting from deep M1 may be the primary target in effective DBS [50].

In contrast to the extensive *in vivo* literature, oscillatory activity in M1 has remained little explored *in vitro*. We have described a pharmacological approach to obtain persistent oscillations in slices of rat M1 [11] and showed that this region will generate synchronous network oscillations preferentially at beta frequency. These oscillations were found to be generated in deep layers and dependent upon fast synaptic inhibition mediated by GABA_A_ receptors. A contribution from NMDA receptors and GABA_B_ receptors was also evident, while electrical coupling of the neuronal network by gap junctions appears necessary for robust rhythmogenesis. These features, including the involvement of glutamate and GABA synaptic drive and direct electrical coupling, are common to many types of oscillatory activity in hippocampus, entorhinal cortex and somatosensory cortex [12], [13], [14], [15], [16].

Field oscillatory activity arises through the synchronous activity of pyramidal neurons. In neocortex, two classes of glutamatergic pyramidal cells, regular spiking (RS) and intrinsic bursting (IB) cells, have been characterised electrophysiologically *in vitro* [17], [18], [19]. Inhibitory GABAergic neurons within the neocortex display extensive electrophysiological and morphological diversity [20], [21], [22], each cell type contributing differently to the pattern of multiple oscillatory frequencies [23], [24]. However, to date there has been no description of the underlying cellular mechanisms of beta oscillatory activity in M1. In this study we have explored the contribution to the field beta oscillation in the primary motor cortex (M1) made by GABAergic IPSPs and action potentials (AP; spike) recorded intracellularly in single M1 layer V pyramidal cells. We report that beta oscillations induced by co-application of kainic acid and carbachol in slices of M1 *in vitro* are mediated by beta-frequency IPSPs acting to control spiking activity in the large pyramidal cells present in layer V of M1. We show that IPSPs in pyramidal cells occur at beta frequency and are highly coherent with the local field potential (LFP) signal, whereas spike activity in the same cells, though highly coherent with beta oscillations, occurs at much lower frequency, indicating that individual pyramidal neurons are active only sparsely during on-going beta activity. Phase analysis of the relationship between APs and the LFP revealed high vector strength at beta frequency, suggesting that, as in other systems [25], [26], motor processing in M1 may depend on both amplitude and phase of cortical oscillatory activity.

## Methods

Extracellular local field potential (LFP) and intracellular (sharp microelectrode) recordings were made from the M1 primary motor cortical region in sagittal slices obtained from 80–120g male Wistar rats. In accordance with Home Office guidelines, animals were maintained in a temperature and humidity controlled environment on a 12/12 light dark cycle and allowed access to food and water *ad libitum*. The cage environment was enriched. All animal procedures were performed in accordance with the Aston University policy on research involving animals and under a project license approved by the Aston University Bioethics Committee. Procedures were also in accordance with the Animals (Scientific Procedures) Act UK 1986 as amended by the European Communities Directive 2010/63/EU. Each rat was terminally anaesthetized with isoflurane (20% in N_2_/O_2_) and transcardially perfused with approximately 100 ml ice-cold sucrose-based artificial cerebrospinal fluid (aCSF) of composition (in mM); 171 sucrose, 2.5 KCl, 10 MgCl_2_, 25 NaHCO_3_, 1.25 NaH_2_PO_4_, 10 glucose, 0.5 CaCl_2,_ 1 ascorbic acid, 2 N-acetyl cysteine, 1 taurine and 20 pyruvate and saturated with 95% O_2_ and 5% CO_2_ at pH 7.3 and 310 mOsm. Indomethacin (45 µM), a cyclo-oxygenase inhibitor, was added to the aCSF to improve cell viability [27] and the antioxidants ascorbic acid (300 µM) and uric acid (400 µM) added as neuroprotectants. After the brain was extracted and placed in ice-cold sucrose-based aCSF, sagittal brain slices (450 µm thick) were cut at room temperature using a HM650 V microslicer (Microm GMBH, Germany) and subsequently stored in an interface chamber filled with oxygenated glucose-based aCSF of composition (in mM) 126 NaCl, 3 KCl, 1.6 MgSO_4_, 26 NaHCO_3_, 1.25 NaH_2_PO_4_, 10 glucose, 2 CaCl_2,_ at room temperature (24°C).

### Electrophysiological recordings

For extracellular recordings, slices were transferred to an interface chamber (Scientific System Design Inc, Canada) and continuously perfused at 1–2 ml/min with glucose-based aCSF. The temperature of the perfusate was maintained at 33–34^o^C using a PTC03 proportional temperature controller (Scientific System Design Inc., Canada). Kainic acid (KA, 100 nM) and carbachol (CCh, 5 µM) were added to the perfusate in order to promote neuronal network oscillatory activity which stabilized over a period of 60 minutes. Local field potential (LFP) recordings were made using borosilicate glass microelectrodes filled with aCSF, of resistance 1–3 MΩ (P-97, Sutter instrument Co, USA). Microelectrodes were placed into layer V and layer II of M1, which was located using a stereomicroscope (Leica Wild M3Z, Leica UK). The M1 region of the cortex was taken as being dorsal to the lateral ventricle and the caudal extent of the striatum, within which layers II (superficial) and V (deep) were identified by visual inspection. Signals were amplified 1000-fold and low-pass filtered using an extracellular amplifier (EXT-02F, NPI, Germany) and conditioned using an 8-pole Bessel filter (LHBF48X, NPI, Germany) to a final gain of ×1000. All signals were also conditioned using a Humbug (Digitimer, UK) to subtract low amplitude mains noise.

Concurrent intracellular recordings were made in Layer V, using borosilicate glass sharp microelectrodes of resistance 50–120 MΩ, filled with KCl (3 M) and 1-trimethylammonio-5-(1-adamantane-methylammoniopentane) dibromide (IEM1460; 5 mM) [28], which blocks AMPA-type glutamate receptor channels in the recorded cell [29]. These sharp microelectrodes were connected to an SEC 05 LX amplifier via a low noise headstage (NPI, Germany), with signals low-pass filtered at 1.3 kHz. All recordings were digitised at 10 kHz using pClamp 10.3 and a Digidata 1440A (Molecular Devices, USA) and analysed with Clampex 10.3, Spike2 software (Cambridge Electronic Design, UK) and KSpectra software (Spectraworks, USA). Graphs were prepared using Graphpad Prism (Graphpad, USA).

### Data Analysis

Data are expressed as mean+standard error of the mean (SEM). Cell input resistance was derived from voltage-current plots over the range −55 to −75 mV. Power spectra were derived from 10 s periods of simultaneous LFP and intracellular recording, with single neurones either held at between −80 to −100 mV with current injection (to eliminate action potentials and to optimise detection of spontaneous depolarising postsynaptic potentials), or examined at resting potential, without any intracellularly applied direct current. The incidence of action potentials (at resting potential) were marked as single events, and used to perform spike-triggered averaging of the LFP signals. The significance of oscillatory data (power spectra and coherence) was ascertained at 99% level using multi-taper method (MTM) analysis performed with KSpectra software (SpectraWorks) run on an Apple iMac. Within KSpectra, resolution was set at 5, with the number of tapers at 9. Peaks in power spectra, or coherence between two signals, were grouped into 3 frequency bands: 3–14 Hz, 15–40 Hz, and 41–100 Hz to facilitate meaningful pooling of data. Data were derived from 27 sets of recordings from a total of 11 different brain slices, all taken from different animals. We chose to measure power in 3 frequency bands (3–14 Hz, 15–40 Hz, and 41–100 Hz), which broadly correspond to theta, beta and gamma bands. Although the precise divisions between such bands vary considerably in the literature and there is no common agreement on terms (e.g. theta versus mu in motor cortex), our recordings of LFPs in layer II/V of M1 *in vitro* (unpublished observations) indicated that these bands bracketed fundamentally different forms of pharmacologically induced oscillatory activity. Data from recordings in which no significant peak in the power spectra in the range 3–100 Hz in any of the LFP or intracellular recordings were excluded from analysis.

### Phase analysis

The LFP data were first filtered using a finite impulse response (FIR) filter centred on the frequency of interest, f_0_, with a pass band f_0_±2 Hz. Phase angle data were calculated by convolving the filtered LFP data with a complex Morlet wavelet function [30] to produce complex time-frequency data, w(t, f_0_), from which amplitude and phase information were extracted. Briefly, the Mortlet wavelet function is defined as 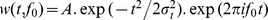, where 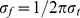, normalisation factor 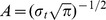 and wavelet bandwidth parameter 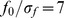. Defining the time of each spike maxima as 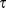
and the filtered LFP phase history as 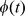, the phase angles of interest were 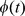. The mean phase vector magnitude 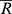, is defined [31] as:



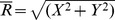
 where 
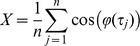


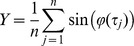
 and n = the number of samples.

As the mean phase angle is not specified, the Rayleigh test may be applied to the vector magnitude 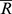, to differentiate between a unimodal distribution of phase angles (indicating phase locking) and a random or uniform distribution of phase angles (indicating no phase locking). For sufficiently sized samples (n>50), the following approximation of the Rayleigh Z formula^31^ may be used, 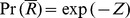, where 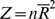. Evidence for a temporal offset in the phase locking mechanism was explored by performing the phase magnitude calculation with a timing offset added to the phase index. Calculations were conducted with offsets ranging between −400 ms and +400 ms with increments of 1 ms. Spike events occurring within the first and last 400 ms of the data set where eliminated from the calculation, which placed an additional condition on the required number of spike events. To ensure a minimum number of spike events (n>50) in the phase vector calculation, only data sets initially comprising at least 70 spikes were included in the analysis. Where results are presented within a graph showing the phase vector magnitude as a function of LFP frequency or temporal offset in spike timing, a correction factor is applied to the vector magnitude significance threshold appropriate to the multiple comparisons in the data and significance level declared.

## Results

### Significant Power in LFPs in beta frequency range

**Figure 1:**
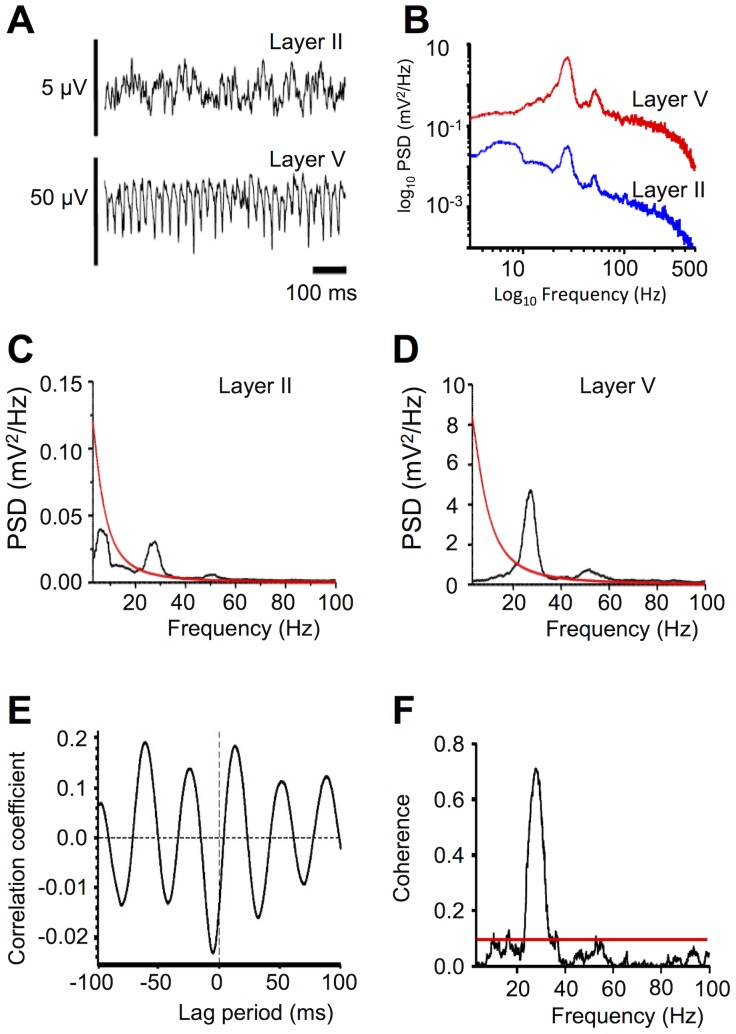
Characteristics of beta activity in local field potentials (LFPs). Layer II and V LFPs show significant power in the beta frequency range, which is both correlated and significantly coherent between layers II and V. (A), sample of filtered recording showing LFPs acquired concurrently in layer II (upper record) and layer V (lower record). (B), power spectra derived from these LFPs, plotted on log scales as power spectral densities (PSD), showing peaks in beta (20–30 Hz) range. (C) and (D), power spectra of same LFPs from layer II and layer V respectively, on linear scales, showing 99% significance levels above red lines. Significant peaks are present at 25.7 Hz (layer II), and at both 26.6 and 51.9 Hz (layer V), with no single clear peak shown in range 40–100 Hz in layer II, although significant power is evident. (E), strong cross-correlation of LFPs from layers II and V, with period of around 34 ms. (F), coherence between LFPs in layer V and layer II (in same recordings as (A) is significant at 99% confidence level (above red line) at 10.3 and 50.3 Hz, but most markedly at 27.6 Hz.

In the M1 slice, beta activity induced by combined application of KA and CCh was observed in both layers II and V in most slices. The amplitude of fast oscillatory activity was always greater in layer V (**Fig. 1A and B** **)**. In layer V, significant (>99% confidence limits) peaks in the power spectra in the range 15–40 Hz, with a single distinct significant peak of mean frequency 29.3+0.9 Hz was seen (n = 18; **Fig. 1D**). Similarly, in layer II significant power between 15–40 Hz, with a single distinct peak seen at 31.8+1.1 Hz was seen (n = 18; **Fig. 1C**). In 4 recordings (3 from the same slice) there was significant power in layer II unaccompanied by that in layer V, while the reverse was true in 3 recordings. In the range 40–100 Hz, significant peaks in the power spectrum were seen in layer V, although in only 7 cases was there a single distinct peak, of mean frequency 60.9+6.1 Hz (n = 7). The amplitude of this peak co-varied with the peak at beta frequency, suggesting that this was a harmonic. In layer II, 5 recordings showed a distinct single peak of mean 68.1+7.6 Hz. In the range 3–14Hz no significant power was seen in layer V, but was present in layer II in 3 instances (2 in the same slice) at 11.6+1.9 Hz (n = 3). Typical power spectral density plots are shown in **Fig. 1C and D**, where beta frequency peaks (and harmonics) can be seen rising above the significance line (red).

Thus significant power in the 15–40 Hz range was commonplace in both layers V (70%) and II (81%), with clearly defined peaks at 29 and 32 Hz respectively displayed in 67% of cases in both layers. Significant power in the range 40–100 Hz was also observed in both layers V (55%) and II (96%), but clearly defined peaks (of 61 and 68 Hz) less frequently seen (only in 26% and 19% of cases respectively), while little power at 3–15 Hz was evident in either layer V or II.

### Coherence and phase-relationship of LFPs in layer V and layer II

Previous studies (e.g. [15]) have reported that oscillatory activity in slice models of persistent oscillatory activity shows characteristic inter-laminar relationships, including strong coherence and phase-reversal between deep and superficial layers, and this was the case in M1. Typically, activity in layer II was phased-reversed with respect to that in layer V, and showed a lag of −6.7+2.1 ms **(** **Fig. 1E** **)**. MTM analysis **(** **Fig. 1F** **)** showed significant (>99% confidence) coherence between LFPs in layer V and layer II, which was detected in the 15–40 Hz range during 23 of 27 recordings made and displayed a distinct peak of 26.8+1.9 Hz in 18 instances. In the range 40–100 Hz, significant coherence was seen in peaks in 22/27 sets of recordings, with a distinct peak displayed at 59.5+4.3 Hz in 15 cases, whereas between 3–14 Hz, only 7 recordings showed any such significant coherence, with a distinct peak shown at 7.8+1.2 Hz in 6 cases. Therefore the oscillations in the LFPs in both layers V and II appear coupled, and coherent at both 27 and 59 Hz in the majority of cases, while coherence at 3–14 Hz was far less evident.

### Cell properties

**Figure 2:**
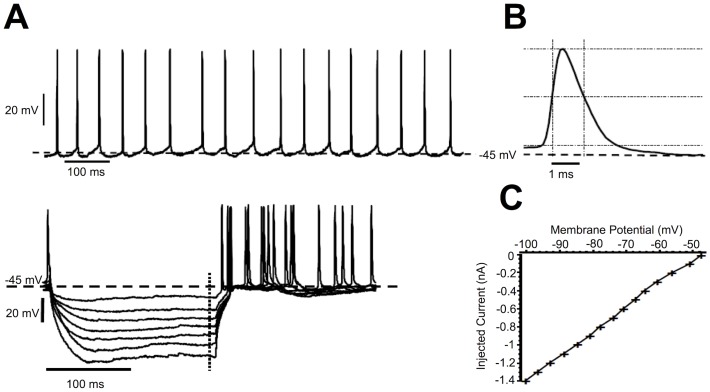
Properties of a layer V pyramidal cell, demonstrated with sharp microelectrode intracellular recording. (A), recording of resting membrane potential, showing spontaneous action potential firing at 18.1 Hz. Right panel: a single action potential on an expanded time scale, with dashed cursor lines indicating method of measuring amplitude (between top and bottom horizontal cursors: 65.3 mV) and duration at ½ maximal amplitude (between vertical cursors: 1.10 ms). (B), superimposed records of membrane potential showing response to successive 200 ms hyperpolarizing pulses of current (not shown) injected in multiples of 0.2 nA from baseline of zero (ie. resting potential). (C), voltage-current plot derived from a series of current pulses injected in multiples of 0.1 nA into the same cell [including those in (B)] in which steady-state voltage attained near end of current pulse [dashed vertical line in (B)] is plotted. Slope of line (best fit in range −55 to −75 mV) yields input resistance value of 45 MΩ. All records from the same cell.

Intracellular recordings made were in slices concomitantly manifesting oscillatory activity. The 27 intracellularly recorded layer V cells used in this study displayed the properties summarised in **Table 1**. Most cells (24/27) spontaneously fired action potentials in the absence of any injected current (‘at rest’), often in a regular manner **(** **Fig. 2A** **)**. Cells with action potentials that did not exceed zero mV at their peak were not accepted for study. Input resistance was measured following a current injection protocol of a series of 200 ms pulses on multiples of 0.2 nA and a typical recording and I–V plot is shown in **Fig. 2B and C**. These cells were of sufficiently uniform character to be considered of a single type, and most likely to be pyramidal cells.

**Table 1:** Basic intracellular properties of recorded cells used in this study.

Property		
Action potential firing rate at rest 1	7.1+1.2 Hz	N = 27
Action potential duration ^2^	1.23+0.10 ms	N = 26
Action potential amplitude	61.0+1.3 mV	N = 26
Input resistance ^3^	59.0+6.3 MΩ	N = 18

^1^ 4 cells were quiescent; ^2^ at ½ maximal amplitude; ^3^ in range −55 to −75 mV.

### IPSPs

**Figure 3:**
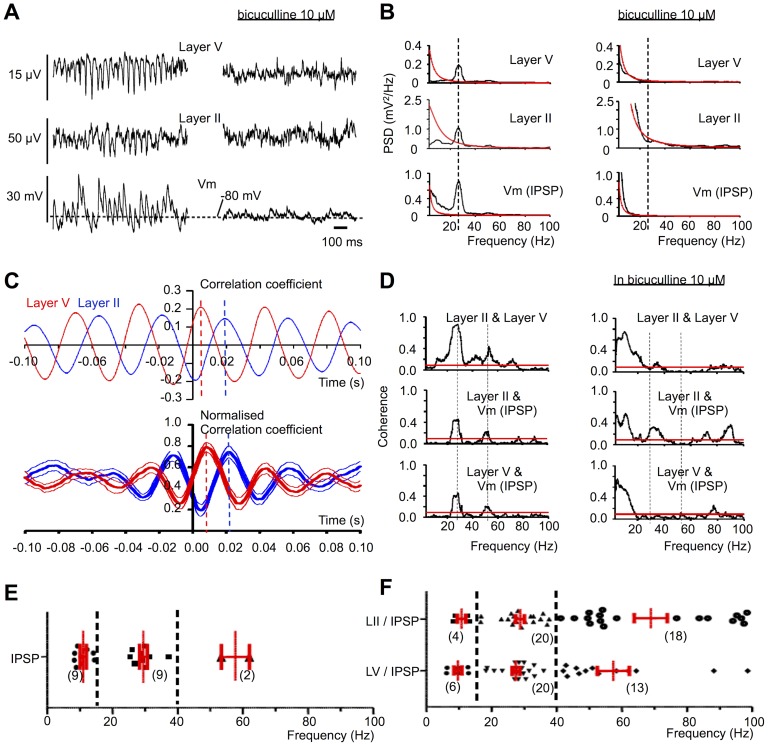
IPSPs in layer V cells are strongly coherent with LFPs in the beta range. (A) Concurrent LFPs from layers V and II, and intracellularly recorded membrane potential (Vm) from a cell in layer V. Oscillations and IPSPs (at −80 mV, optimised for IPSPs) are blocked following application of GABA_A_ receptor antagonist bicuculline (right panel). (B) Power spectral densities (PSD) of LFPs from layers V and II, and of Vm (with IPSPs), showing 99% significance levels (above red lines) at beta frequencies, and harmonics thereof, which (right panels) are blocked by bicuculline. Vertical dashed lines indicate 27 Hz for reference. Same recordings as (A). (C), upper panel: cross-correlograms of LFPs from layers II (blue) and V (red) with Vm from same recordings as in (A) and (B). Lower panel: normalised, cross-correlated data (means+SEM) between Vm (displaying IPSPs) and LFPs in layer V (red) and layer II (blue) pooled from all 20 recordings showing significant IPSP-LFP coherence in 15–40 Hz range. The IPSP leads layer V peak (red dashed line) by 7.2 ms and layer II peak (blue dashed line) by 20.5 ms. (D) Left column: coherence between each of layer II and layer V LFP (top row), layer II LFP and IPSPs (middle row), and layer V LFP and IPSPs (bottom row) in each case demonstrates single significant (>99%) peaks at beta frequencies, and harmonics thereof, which is abolished by bicuculline (right panels). Same recordings as A, B and C. (E) and (F), data pooled from all recordings within 3 frequency ranges (demarked by vertical dashed lines, with mean ± SEM in red, n in parentheses) showing (E) the distribution of the single largest significant (>99%) power spectrum peaks for Vm (optimised for IPSPs), and (F) peak frequencies of coherence between LII, LV, and Vm (IPSPs).

On hyperpolarizing membrane potential to the range −80 to −100 mV, transient depolarising events, increasing in amplitude with increased membrane hyperpolarisation, were clearly observed in 24 of the 27 cells examined (**Fig. 3A** **).** These were taken to be GABA-mediated spontaneous inhibitory postsynaptic potentials (IPSPs), rendered depolarising by chloride loading of the cell with KCl from the recording electrode. The amplitude of both the IPSP and the LFP was profoundly depressed by application of the GABA_A_ antagonist bicuculline methiodide (10 µM), indicating that (i) there was little, if any, contribution of glutamate receptor activation to these events, and (ii) GABA_A_ receptor-mediated inhibition was responsible for both the IPSPs and the field oscillatory activity, which was also blocked by bicuculline (as previously reported^11^). Spectral analysis of membrane potential recordings made in the range −80 to −100 mV revealed significant (>99% confidence limits) power in the range 15–40 Hz in 23/27 cells, with a single distinct significant peak frequency in 9 of these cells of 29.5+1.3 Hz (**Figs. 3B and E**). In the 40–100 Hz range, significant power was seen in 21/31 layer V cells, but with clearly distinct single peaks in only 2 (mean 57.7 Hz). In the range 3–14 Hz, significant power was seen in 13/31 layer V cells, with a distinct peak at 11.0+1.0 Hz in 9 cells **(** **Fig. 3E** **)**.

### Coherence and Cross-Correlation Between IPSPs and LFPs

Membrane potential recordings manifesting IPSPs in layer V cells showed significant coherence with Layer V LFPs in the 15–40 Hz range in 23 of 27 instances, with a distinct peak at 27.5+1.2 Hz shown in 20 cases. Significant coherence between IPSPs and the layer II LFP was also observed in the 15–40 Hz range in 21 of 27 recordings, with a distinct peak at 28.7+1.3 Hz (n = 20). In the 40–100 Hz range coherence was seen with the layer V LFP in 20 of the 27 recordings, with a distinct peak at 57.3+5.1 Hz (n = 13), and with the layer II LFP in 19 recordings with a distinct peak at 69.0+5.2 Hz (n = 18). In the 3–14 Hz range such coherence with layer V LFP was seen in only 7 of the 27 recordings, with a distinct peak at 9.7+1.3 Hz (n = 6), and with the layer II LFP in 4 cells at 10.8+1.5 Hz (**Fig. 3D and F**). Such coherence was blocked by bicuculline (10 µM; **Fig. 3D**). Cross-correlation of both the layer V and layer II LFP with membrane potential (while held at −80 to −100 mV, thus optimised for IPSPs) indicated a clearly phase-locked correlation, with the IPSP leading the peak of the layer V LFP by 7.2 ms (based on n = 20 cells), and the peak of the layer II LFP by 20.5 ms (**Fig. 3C**).

In summary, the highest incidence of significant power in IPSP recordings was seen in layer V cells at 29 Hz. Moreover, coherence of IPSPs in layer V cells with LFPs, at mean frequencies of 28 and 57 Hz (layer V) and 29 and 69 Hz (layer II), was seen in the majority of cells, and this is consistent with the LFP power in this beta range, and likely the first harmonic thereof, being driven by the IPSPs.

### Spikes

**Figure 4:**
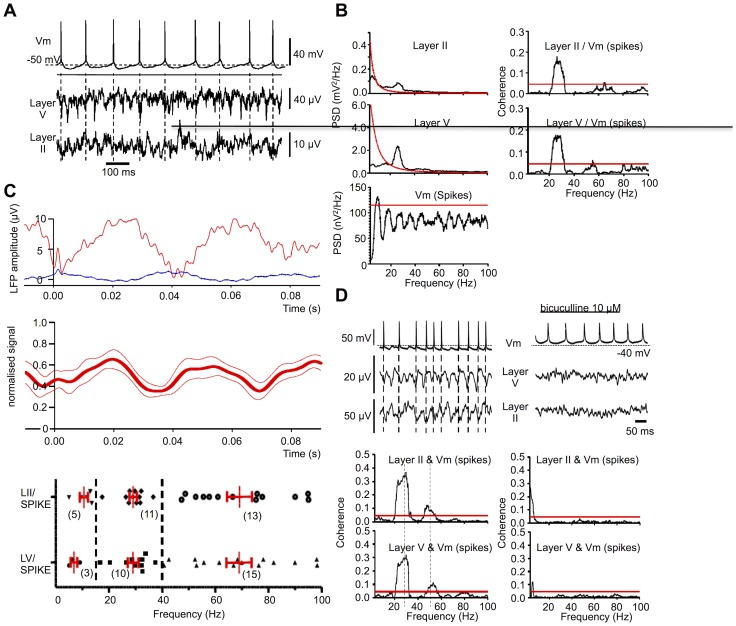
Layer V pyramidal cells action potentials are coherent with and phase-locked to LFPs. (A) membrane potential (Vm) recording (without DC current injection), with spontaneous action potentials (spikes), together with concurrent (unfiltered) records of LFPs. (B), left panels: Power spectral densities in LFPs are significant (99% above red lines) in beta range, but for Vm are close to spontaneous spike firing rate. Right panels: coherence between Vm and layers V and II LFP is seen in beta range, and harmonics thereof (same recordings as A). (C), upper panel: spike-triggered averages of LFPs from layer V (red) and II (blue), time-locked to each of 84 spikes occurring over a 10 s period (at t = 0 on x-axis). Spikes precede by 2–3 ms the trough, and peak, of the layer V and II oscillations respectively, both of which display a period of around 40 ms. Taken from same cell as in A and B. Lower panel: pooled, normalised, layer V LFP spike-triggered average data (mean ± SEM) from all the 10 recordings that showed significant coherence in the 15–40 Hz range between layer V LFP and Vm (during spontaneous firing). The layer V LFP peak follows the spike by approximately 20 ms. (D), upper panels: Records of (Vm) recorded at rest, during spontaneous spike firing, and of layer V and II LFPs, in absence (left) and presence (right) of bicuculline (10 µM). While spikes persist in bicuculline, LFP oscillations are abolished. Lower panels: significant beta range coherence between Vm and both layer V and II LFP (left) is abolished in bicuculline (right). Different preparation from panels A–C. (E), data pooled from all recordings showing the distribution of frequencies at which significant coherence between LFPs in layer II and layer V, and spikes, was detected, grouped into 3 frequency bands (mean ± SEM in red, n in parentheses).

It was apparent from raw data records that action potentials (spikes) in spontaneously firing cells often corresponded to the troughs of the layer V LFP, and to the peaks of layer II LFP (**Fig. 4A and D**); this relationship was explored further. Frequency analysis of membrane potential recordings from the 23 spontaneously firing neurones (with mean firing rate of 8.4+1.2 Hz) showed a significant (>99%) peak(s) in the power spectrum for 10 cells. These peak frequencies varied between 8.5 and 52.4 Hz (10.8+1.0 Hz, n = 7, in 3–14 Hz range; 20.8+2.1 Hz, n = 8, in 15–40 Hz range). The lowest significant peak in any given cell was 93.7+4% (n = 10) of the firing rate measured over the 10 s period analysed, indicating a considerable regularity in the firing pattern of these 10 cells (a ratio of 100% would indicate a constant inter-spike interval). Moreover, in 6 of these 10 cells a second significant peak in the power spectrum was seen at 200.6+4% (n = 6) of the lower peak, probably reflecting harmonics in the power spectrum. Thus the power in the resting membrane potential records arose largely from action potentials.

### Relationship between Spikes, Membrane Potential and LFPs: Coherence Analysis, and Spike-Triggered Averaging

Significant (>99%) coherence between membrane potential of spontaneously firing neurones and the layer V LFP was observed in the 15–40 Hz range in 10/23 cells examined, with a distinct peak at 29.0+2.1 Hz (n = 10). Significant coherence with layer II LFP was also observed in the 15–40 Hz range in 11/23 cells, with a distinct peak seen at 27.8+2.0 Hz (n = 11). In the 40–100 Hz range, such coherence with the layer V LFP was seen in 21/23 cells, with distinct peak at 69.0+4.9 Hz in 15 cases, and with the layer II LFP in 16 cells, with a distinct peak at 69.2+4.9 Hz in 13 cases. In the 3–14 Hz range, coherence with the layer V LFP was seen in only 3/23 cells, at 6.9+1.5 Hz (n = 3), and with the layer II LFP in 5 cells, at 10.0+2.1 Hz (**Figs 4B and E**). The incidence of spike-LFP coherence was greater than that of cells showing significant power in the frequency analysis of spike trains alone. This was strongest between spike trains and both layer V LFPs (around 29 and 69 Hz) and layer II LFPs (around 28 and 69 Hz), despite the mean firing rates being only 8.4 Hz (around which there was little such coherence). While this indicates a contribution from layer V cell firing to generating the predominant oscillation in both layers V and II, it does not indicate (as seen with the IPSPs) a likely 1:1 correspondence of spike firing to the 28 Hz beta frequency cycle manifest in the LFP recordings.

The relationship between spontaneous spikes and the LFPs was explored using spike-triggered averaging (**Fig. 4C**). This demonstrated that spikes were time-locked to the LFP, preceding the peak of the layer V LFP beta oscillation by approximately 20 ms (**Fig. 4C**, lower panel, based on data pooled from 10 cells). Assuming a mean predominant oscillation frequency of 28 Hz, this period of 36 ms indicates the spikes, when they occur, tend to do so around 2 ms prior to the trough of layer V LFP and – assuming antiphase in layer II – around the peak of the layer II LFP. While LFP oscillations were abolished by bicuculline, as was coherence between membrane potential and LFP in both layers V and II (**Fig. 4D** **)** lower panels), spike firing itself was not bicuculline-sensitive, and thus clearly not dependent on any GABA-ergic synaptic input.

### Relationship between Spikes and LFPs: phase angle analyses

**Figure 5:**
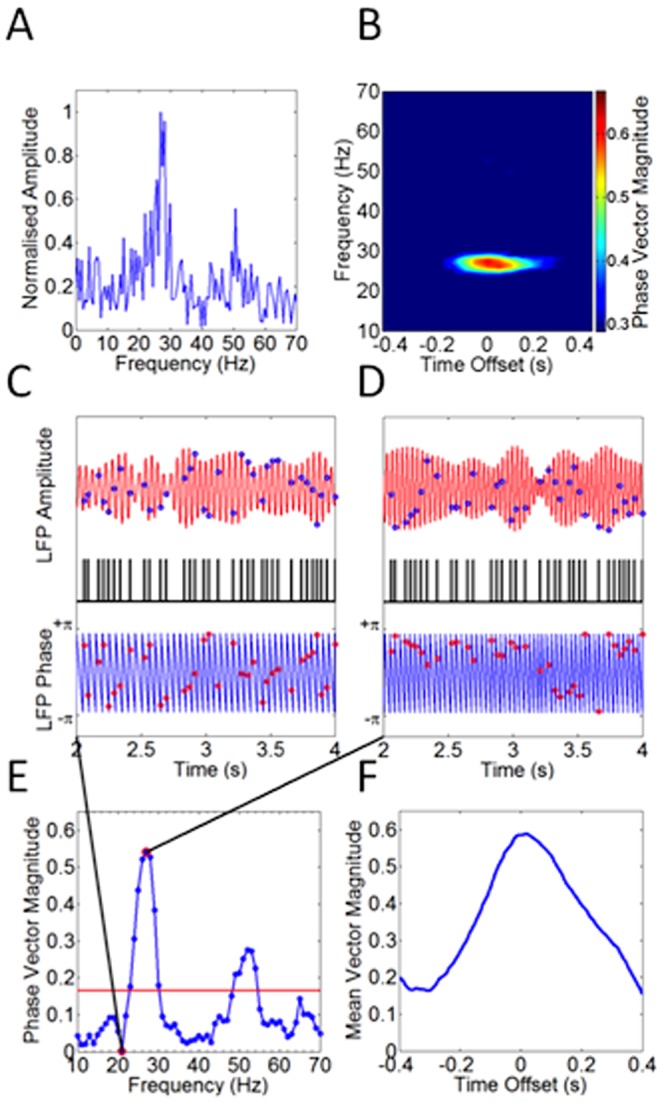
Spike firing is strongly phase-locked to the Layer V LFP. A) Fourier transform representation of the unfiltered LFP shows significant energy is present in the signal up to 70 Hz. (B) Data presented in A are expanded to show phase locking statistics for frequencies 10–70 Hz and time offsets −400 ms to +400 ms. Phase locking data not reaching significance (*P*<0.01) are displayed dark blue. The Rayleigh Z significance threshold is corrected for the multiple frequency and time offsets displayed. Data from a single spike train/LFP recording. (C) A three second data segment showing distribution of spike train (middle record, black) - indexed measurements in both amplitude (upper record, red) and phase (lower record, blue), representing LFP data band - filtered at 21 Hz. Randomly distributed phase values (red circles) show that, at this frequency, the spike train and LFP are not phase aligned. (D) Using the LFP data filtered at 26 Hz, there is a distinct clustering of phase values indicating strong phase locking between spike train and LFP. (E) Plotting mean phase vector magnitude values against frequency shows two regions of phase locking, at 26 Hz and 51 Hz. The red line shows the significance threshold at p<0.01. Same data set as A and B. (F) The timing relationship between spike train and 26 Hz filtered LFP is investigated by calculating the mean phase vector magnitude at different temporal offsets between spike train and filtered LFP. The graph indicates that the spike train is most strongly phase locked to the LFP after a delay of 18 ms for this data set.

The coherence and spike-triggered averaging described above may be compromised by the influence of the amplitude of oscillations on the overall measurements of coherence. In order to mitigate this problem, and also to determine the precise relationship between spiking and the LFP, we further analysed the interactions between spikes and the LFP in layer V through a phase-angle based approach. Fourier analysis of beta activity **(** **Fig. 5A** **)** showed significant energy in the LFP to justify performing phase measurements over the frequency range of 10 Hz to 70 Hz. Of 17 datasets analysed, 9 showed phase locking between spike train and LFP at a significance level of *P*<0.01 (Rayleigh Z statistic). An example Rayleigh Z statistical analysis for a data set showing significant phase locking history is shown in **Fig. 5B** **.**

Evidence for phase locking interactions between spike train and layer V local field potentials was explored further by analysing the distribution of a subset of phase angles within band-filtered LFP data, specifically those phase data indexed by the timing of spike train maxima [32]. Spike train indexed phase angles were averaged, producing a phase vector magnitude ranging in value between 0 and 1. Thus phase vector magnitude is a measure of the strength of phase locking present with a value of 0 indicating no phase locking between spike train and LFP, while perfectly synchronized noiseless data would yield a phase vector magnitude of 1.

As **Fig. 5C** shows, with 21 Hz filtered LFP data, the relationship with the corresponding spike train does not suggest phase-alignment. However, using data band-filtered at 26 Hz, a distinct clustering in phase values was seen **(** **Fig. 5D** **)**, indicating strong phase locking between spike train and LFP. Plotting mean phase vector magnitude values **(** **Fig. 5E** **)** across frequency showed phase locking, at 26 Hz (*P*<0.01). Finally, calculation of the mean phase vector magnitude at different temporal offsets **(** **Fig. 5F** **)** indicated that the spike train was most strongly phase locked to the LFP that occurs after a delay of 18 ms.

These data indicate that whilst spiking frequency is much lower than LFP beta frequency in deep layers of M1, such activity is strongly phase locked to the LFP, almost certainly dictated by the characteristics of the bicuculline-sensitive beta frequency IPSP described above.

## Discussion

In our previous study [11], it was shown that phasic inhibitory and phasic/tonic excitatory synaptic inputs were required for persistent beta oscillations in M1 layer V coronal slices *in vitro*. In this study we have shown that, 1) in slices, persistent beta oscillatory activity can be simultaneously evoked in layers V and II of M1, 2) beta frequency activity is strongly correlated between LII and LV and with the intracellularly recorded IPSP in layer V, 3) regular spiking, presumably principal, cells in M1 fire action potentials at low frequency during on-going network oscillations, and 4) despite their relatively low frequency, spikes in layer V cells are phase locked to the LFP.

The layer V cells from which intracellular recordings were made have properties resembling those previously described as ‘regular-spiking’ (RS) pyramidal cells [17], [18], [19]. In contrast to these previous studies, no ‘intermittent burster’ (IB) cells were encountered. While this may just reflect a sampling bias, it may be that bursting behaviour was not favoured by the temperature of the experiments [33], and/or the (depolarizing) actions of the kainic acid and carbachol used to induce beta oscillations.

The precise nature of the mechanisms underlying the LFP remain elusive, however a dominant role of synaptic currents over action potentials in the contribution to low frequency (<100 Hz) rhythmic activity seems likely [48], [49]. Hence, it is probable that the principal current source underlying the peak of the layer V LFP is the hyperpolarizing IPSP in pyramidal cells in layer V. A parsimonious simple local circuit model would have the IPSP arising from a local interneuron network whose output is entrained by mutual inhibitory connections, and also by EPSPs originating in pyramidal cells, following their action potentials. Spike firing in pyramidal cells would then aid entrainment of network activity and strengthen the oscillation. Making this assumption, the antiphasic relationship of the layer II LFP to the layer V oscillation probably reflects activity in layer V cell dendrites known to course through layers I-III acting as current sinks, rather than any contribution from layer II neurones to generating the beta oscillation, hence, a current sink-source dipole in deep and superficial layers would appear to be the simplest explanation for our observations.

The characteristic frequency of the oscillations observed here - around 29 Hz – places them at the upper end of the beta frequency band, generally taken to range from 13 Hz up to 30, 35 or even 40 Hz, depending on species [34]. Although this oscillatory frequency is similar to the beta2 (20-30 Hz) oscillations observed in layer V of rat somatosensory cortex [16] the two activities appear pharmacologically and mechanistically different. In M1 *in vitro*, beta oscillatory activity requires muscarinic M1 receptor activation, is insensitive to AMPA receptor blockade, but extremely sensitive to GABA_A_ receptor modulators such as pentobarbital, zolpidem [11] and beta carbolines (10 nM, unpublished observations), as well as being blocked by bicuculline. As in M1, beta2 activity in somatosensory cortex is insensitive to AMPA receptor blockade, however, these oscillations appear to be relatively insensitive to GABA_A_ receptor blockade and show intense spikelet activity in IB cells at beta frequency [16], which is not observed in M1.

The low firing frequency of pyramidal cells, phase-locked with faster field activity, has been previously reported in gamma oscillations [14], [15]. Synaptic mechanisms appear to account for the discrepancy between field and individual neuronal activity [14] and this type of mechanism may also apply in M1. In present study, EPSPs during beta oscillatory activity had been blocked in the individual cells recorded, and could not be examined directly. However, any contribution from phasic recurrent excitatory inputs from pyramidal cells to both pyramidal cells and interneurons in synchronising pyramidal cell output may have been overridden by the strong tonic excitation of all cell types likely to result from activation of both kainate and muscarinic M1 receptors. Alternatively, the longer decay time of IPSPs (compared to decay time of IPSPs seen during gamma oscillations) may shunt EPSP activity. In addition, action potential firing may only occur when EPSPs coincide with activation of an intrinsic membrane conductance (for example, activation of I_h_, Ca_V_ 3.1 calcium current or gap junction mediated spikelets). In support of this possibility, intrinsic properties of postsynaptic neurons have been suggested to interact effectively with GABAergic inputs in shaping supra-threshold activity [35]. In our experiments, recordings were performed with electrodes filled with a solution designed to block AMPAR and load cells with chloride, and hence the precise relationship between spike timing and IPSPs may be subtly altered compared to the situation in which IPSPs are not reversed. Overall, it is likely that GABA-ergic interneurons in M1 [36] receive IPSPs at beta frequency, and provide phasic GABA_A_ receptor-mediated inhibitory inputs at beta frequency to large populations of RS cells in M1. Alternatively, another interneuron network could be generating the beta frequency activity. This network would be required to make synaptic or electrical contact with principal cells and/or FS cells. One such candidate is the low-threshold spiking (LTS) cell [37], [38], [39] which preferentially targets distal apical dendrites [35], [38] and which have been shown to undergo oscillatory activity in the beta range by a number of groups [44], [45], [46]. In a recent computational modelling study [47], distal dendrite targeting ‘Martinotti’ cells have been proposed to control layer V neocortical pyramidal cell spiking in the 5–30 Hz range through oscillatory inhibition, which serves to allow distal dendritic excitation to drive somatic spiking. By contrast, asynchronous distal dendritic inhibition may facilitate more irregular burst firing. The strong oscillatory activity in our *in vitro* preparation may thus explain why we were unable to see intrinsic bursts firing patterns in the pyramidal cell recordings made.

The activity of each RS cell only contributes to at most every second beta cycle. Thus, the population of RS cells mediating any given cycle will be highly variable. This may allow dynamic formation of a network though the recruitment of neurons to a phase-coherent population. In this way, neuronal pools within a pre-existing population of excited pyramidal cells in the beta oscillating network could be selected or suppressed by newly arriving, phase altering, motor or sensory information. Changes in phase synchrony will be reflected in alterations in beta power, and thus may have relevance in PD, where beta power is enhanced. However, the contribution of RS cells to the population mediating the beta oscillation may also be related to alterations in the number of neurons participating in ongoing activity or changes in the underlying frequency of spiking in principal neurons. The low-frequency but phase locked spikes in layer V with the LFP corroborates previous reports of sparse coding activity in cortical structures [24], [40]. Interestingly, there is some suggestion in our data **(** **Fig. 5D** **)** that phase locking may occur in more than one mode, and this is the subject of current investigations.

Beta oscillations in basal ganglia–motor cortical loops have been proposed to facilitate maintenance of ongoing sensorimotor status [41], although this has been modified recently by the proposal that its role is to gate access of novel cues (distractors) to motor programmes, permitting appropriate task selection and prosecution on the basis of existing cues [42]. In the intact human M1, we have previously reported neuroplastic effects of transcranial stimulation on beta activity and its effects on control of voluntary movement [43]. The preparation and methodology described here may be useful in future studies of beta oscillation of the motor system, and in better understanding of the pathology of neural circuit function in akinetic disorders where beta oscillations predominate.
